# Draft Genome Sequence of *Bradyrhizobium japonicum* Is-1, Which Is Incompatible with *Rj*_2_ Genotype Soybeans

**DOI:** 10.1128/genomeA.01219-15

**Published:** 2015-10-22

**Authors:** Tomoyuki Sakata, Yu Kanesaki, Hirofumi Yoshikawa, Hirohito Tsurumaru, Takeo Yamakawa

**Affiliations:** a Graduate School of Bioresource and Bioenvironmental Sciences, Faculty of Agriculture, Kyushu University, Fukuoka, Japan; b NODAI Genome Research Center, Tokyo University of Agriculture, Tokyo, Japan; c Department of Bioscience, Tokyo University of Agriculture, Tokyo, Japan; d Graduate School of Life Science, Tohoku University, Miyagi, Japan; e Department of Biosciences and Biotechnology, Faculty of Agriculture, Kyushu University, Fukuoka, Japan

## Abstract

We report the draft genome sequence of *Bradyrhizobium japonicum* Is-1, which is incompatible with *Rj*_2_ genotype soybeans. The estimated genome size of this strain is 8.9 Mb. Genome sequence information of this strain will help to identify a causal gene for this incompatibility.

## GENOME ANNOUNCEMENT

*Bradyrhizobium japonicum* is known to form nodules on the roots of soybean (*Glycine max*). However, nodulation of *B. japonicum* strains Is-1 and USDA 122 is restricted by some soybean cultivars (e.g., Hardee) (1–3). This nodulation incompatibility is due to the *Rj*_2_ gene in soybean. Yang et al. revealed that this gene encodes members of the Toll-interleukin receptor/nucleotide-binding site/leucine-rich repeat (TIR-NBS-LRR) class of plant resistance proteins (4). In contrast, a causal gene in the incompatible *Bradyrhizobium* strains has not been revealed (2, 3).

Genomic DNA of *B. japonicum* Is-1 was extracted by using the Wizard Genomic DNA purification kit (Promega, Madison, WI, USA), and fragmented to 400 bp using the Covaris S2-A system (Covaris, Woburn, MA, USA). A library for sequencing was prepared by a NEBNext DNA library prep master mix set for Illumina (New England Biolabs, Ipswich, MA, USA), and paired-end sequenced (2 × 302 bp) on a MiSeq sequencer using a MiSeq version 3 reagent kit (Illumina KK, Tokyo, Japan). Raw reads were trimmed and *de novo* assembled using CLC Genomics Workbench version 7.5.1 (Qiagen, Valencia, CA, USA). Parameters for the trimming were as follows: ambiguous limit, 2; quality limit, 0.001; number of 5′ terminal nucleotides, 10; number of 3′ terminal nucleotides, 40. Parameters for the *de novo* assembly were as follows: mapping mode, map reads back to contigs (slow); update contigs, yes; bubble size, 600; minimum contig length, 1,000; automatic word size, 51; perform scaffolding, yes; auto-detect paired distances, yes; mismatch cost, 2; insertion cost, 3; deletion cost, 3; length fraction, 0.8; similarity fraction, 0.95.

The draft genome of *B. japonicum* Is-1 was assembled into 109 contigs, with an accumulated length of 8,983,878 bp (*N*_50_ = 358,317 bp) and an average GC content of 64.0%. The genome was annotated by the Prokaryotic Genome Annotation Pipeline (PGAP version 2.10), and a total of 8,066 coding sequences (CDSs), 3 rRNAs, and 50 tRNAs were predicted.

Tsukui et al. reported that disruptants of type III secretion (T3SS) structural genes in the incompatible *Bradyrhizobium* strains overcame the nodulation restriction of *Rj*_2_ genotype soybeans (2). This result indicates that a causal gene in the incompatible *Bradyrhizobium* strains may encode a T3SS effector protein. The genome sequence of *B. japonicum* Is-1 will facilitate the identification of this causal gene.

### Nucleotide sequence accession number.

This whole-genome shotgun project has been deposited at DDBJ/EMBL/GenBank under the accession number LGUJ00000000.
